# Prolongierte Ruhigstellung mittels Fixateur externe bei subtalarer Luxation ohne Fraktur: Ein Fallbericht

**DOI:** 10.1007/s00113-025-01583-w

**Published:** 2025-06-02

**Authors:** Yasmin Youssef, Volker Schöffl, Gordian Weber, Steffen Röttel, Christian Willy, Falko Patzsch

**Affiliations:** 1 Klinik für Orthopädie, Unfallchirurgie und plastische Chirurgie, Universitätsklinikum Leipzig, Liebigstr. 20, 04103 Leipzig, Deutschland; 2 Klinik für Orthopädie und Unfallchirurgie, Zentrum für Interdisziplinäre Sportmedizin, Klinikum Bamberg, Buger Straße. 80, 96049 Bamberg, Deutschland; 3 Klinik für Unfallchirurgie, Orthopädie und septisch rekonstruktive Chirurgie, Bundeswehrkrankenhaus Berlin, Scharnhorststr. 13, 10115 Berlin, Deutschland; 4 Docortho MVZ GmbH, Friedrichstraße 94, 10117 Berlin, Deutschland

**Keywords:** Sprunggelenk, Distorsion, Luxation, Fixateur externe, Ligamentärer Schaden, Ankle joint, Sprain, Dislocation, External fixator, Ligamentous injury

## Abstract

**Hintergrund:**

Subtalare Luxationen (simultane Luxationen vom Talokalkanear- und Talonavikulargelenk) ohne begleitende Fraktur sind selten und machen 1–2 % aller Luxationen aus. Die Versorgung wurde in mehreren Fallbeispielen beschrieben und besteht vorrangig aus einer geschlossenen Reposition unter Anästhesie mit anschließender Ruhigstellung.

**Fall:**

Es wird über einen 30-jährigen Patienten berichtet, der sich beim Klettern eine mehrdimensionale nichtfrakturierte Luxation des Sprunggelenks mit vorwiegend subtalerer Komponente (Luxatio subtalolateralis) zuzog. Eine geschlossene Reposition war, wie sich im späteren Verlauf herausstellte, wegen tendinöser Interposition der Sehnen des M. tibialis posterior und des M. flexor digitorum pedis longus nicht möglich, sodass eine offene Reposition durchgeführt werden musste. Erst im Rahmen der operativen Exploration konnte das volle Ausmaß der Verletzung beurteilt werden. Der Patient wurde für 12 Wochen im Fixateur externe ausbehandelt. Danach wurde mit einer krankengymnastischen Beübung begonnen. Bereits nach 6 Monaten postoperativ zeigte der Patient eine gute Mobilität, Funktionalität und Belastbarkeit im betroffenen Sprunggelenk. Nach 30 Monaten zeigte sich der Patient beschwerdefrei.

**Zusammenfassung:**

Die in diesem Fallbericht durchgeführte prolongierte Ruhigstellung nach subtalarer Luxation, mittels Fixateur externe für insgesamt 12 Wochen zeigte langfristig sehr gute funktionelle Ergebnisse.

## Abstract (en)

**Background:**

Subtalar dislocations (simultaneous dislocations of the talocalcaneal and talonavicular joints) without concomitant fracture are rare and account for 1–2% of all dislocations. The treatment has been described in several case reports and consists primarily of closed reduction with the patient under anesthesia followed by immobilization.

**Case:**

A 30-year-old male patient sustained a multidimensional nonfractured dislocation of the ankle joint with a predominantly subtalar component (luxatio subtalolateralis) while climbing. As it turned out later, a closed reduction was not possible due to interposition of the tendons of the posterior tibialis muscle and the flexor digitorum pedis longus muscle, so that an open reduction had to be performed. The full extent of the injury could only be assessed during the surgical exploration. The patient was treated in an external fixator for 12 weeks. Subsequently, physiotherapy was initiated. After only 6 months postoperatively the patient showed good mobility, functionality and resilience in the affected ankle joint. After 30 months the patient was free of symptoms.

**Summary:**

The prolonged immobilization after subtalar dislocation using an external fixator over a total period of 12 weeks, as performed in this case report, demonstrated good long-term functional outcomes.

## Einleitung

Indoor-Klettern und Bouldern sind Sportarten, die zunehmend an Beliebtheit gewinnen und vergleichbare Verletzungsmuster aufweisen [1–5]. Neben Verletzungen an der oberen Extremität sind Verletzungen der Sprunggelenke häufig [1, 2, 5]. Dabei konnte gezeigt werden, dass Verletzungen der unteren Extremität im Vergleich zu Verletzungen der oberen Extremitäten häufiger durch Stürze oder Sprünge von der Kletterwand verursacht werden und mit einer signifikant höheren Verletzungsschwere verbunden sind [5]. In den letzten Jahren hat sich sowohl beim Profi- als auch beim Freizeit-Bouldern eine deutliche Verschiebung hin zu komplexeren Bewegungsabläufen vollzogen. Ebenso zeigt das moderne Wettkampf-Bouldern zunehmend häufiger komplexe Fußverletzungen durch „Fehltritte“ bei „Run-and-Jump“-Bewegungen [6]. Weiterhin zeigen Kletterer, die Kletterschuhe mit starkem Downturn tragen, ein signifikant höheres Risiko für schwerwiegendere Verletzungen [5].

In diesem Fallbericht wird über einen 30-jährigen männlichen Patienten mit einer komplizierten, mehrdimensionalen Luxation des oberen und unteren Sprunggelenks, inklusive des Talonavikulargelenks und deren operative Behandlung sowie postoperative Nachbehandlung berichtet.

## Fallbericht

### Anamnese

Vorgestellt wird ein 30-jähriger Patient, der beim Indoor-Klettern gestürzt ist und sich dabei eine isolierte Verletzung des linken Fußes/Sprunggelenks zugezogen hat. Bei Einweisung in die Rettungsstelle bestand bei dem Patienten ein GCS von 15.

Im späteren stationären Verlauf berichtete der Patient, dass er beim Klettern von einem schmalen Tritt abgerutscht und dabei ins Seil gefallen sei; der Sicherungshaken habe sich ca. 2 m tiefer befunden, wodurch es letztlich zu einem direkten Anprall des Fußes ca. 4 m unterhalb der Absturzstelle an die Kletterwand kam. Während des Klettervorgangs trug der Patient sehr enge, spezielle Kletterschuhe mit Downturn (Absenkung des Vorfußes im Vergleich zum restlichen Schuh), Vorspannung (konkave Form der Fußsohle in Längsrichtung) und Asymmetrie (Form des Schuhs, die zu einer Innenrotation des Vorfußes führt).

### Befund und Diagnose

**Abb. 1:**
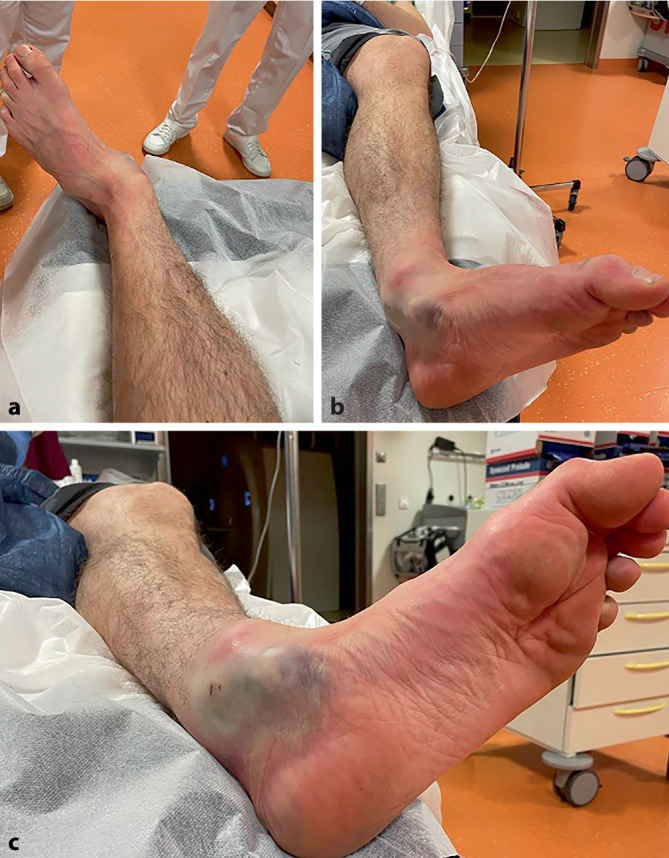
Inspektorischer Befund des linken Fußes bei Ankunft in der Notaufnahme. Deutliche Fehlstellung des Fußes nach lateral mit prominenter Vorwölbung des Talus medial

Bei Eintreffen in der Rettungsstelle zeigte sich eine deutliche Fehlstellung im linken Sprunggelenk mit Versatz des Fußes nach lateral (Abb. 1). Das Sprunggelenk war geschwollen, hämatomverfärbt und druckdolent. Die Beweglichkeit im Sprunggelenk wurde wegen der offensichtlichen signifikanten Fehlstellung nicht überprüft. Die Zehen befanden sich in einer kontrakten Krallenstellung. Die Weichteile des Vorfußes und des Sprunggelenks zeigten Schürfwunden, Kontusionsmarken (Weichteilschaden Grad I nach Tscherne und Oestern) sowie Zeichen einer partiellen Minderdurchblutung im Bereich des Sprunggelenks medialseitig. Die periphere Durchblutung (A. dorsalis pedis mit kräftigem Puls; periphere Rekapillarisierungszeit < 2 s) und Sensibilität waren bis auf eine leichte Hypästhesie im Bereich der 2. bis 4. Zehe intakt.

**Abb. 2:**
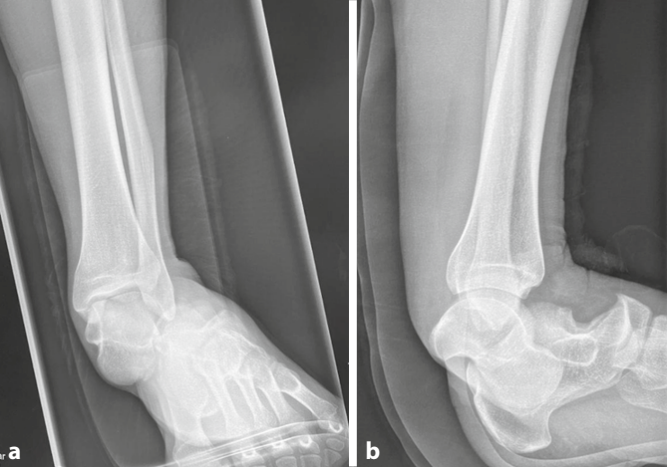
Röntgen des linken Sprunggelenks in 2 Ebenen: komplette Luxation des Kalkaneus nach ventrolateral. **a** Röntgenbild des Sprunggelenks in ap, **b** Röntgenbild des Sprunggelenks seitlich

Im Rahmen der Röntgendiagnostik (Röntgen des linken oberen Sprunggelenks in 2 Ebenen) zeigte sich bei eingeschränkter Beurteilbarkeit (Aufnahme in Behelfstechnik und mit anliegender Schiene) eine vollständige Luxation des Kalkaneus nach ventrolateral (Abb. 2). Die Röntgendiagnostik gab keinen Anhalt für eine Fraktur.

Nach klinischer Untersuchung und bildgebender Diagnostik wurde entschieden, eine möglichst rasche Reposition unter Analgosedierung und Relaxation in der Notaufnahme durchzuführen. Da dies frustran verlief, wurde die Indikation zur umgehenden offenen Reposition unter OP-Bedingungen und Vollnarkose gestellt. Auf die Anfertigung einer präoperativen Computertomographie musste aufgrund der potenziell gefährdeten Durchblutung aus Zeitgründen verzichtet werden.

### Therapie

**Abb. 3:**
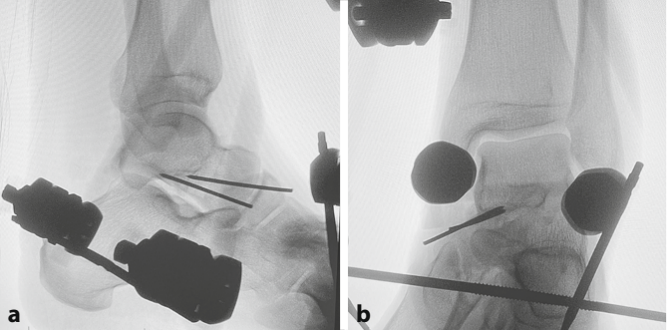
Intraoperative Röntgenkontrolle. **a** Röntgenbild des Sprunggelenks seitlich, **b** Röntgenbild des Sprunggelenks in ap

**Abb. 4:**
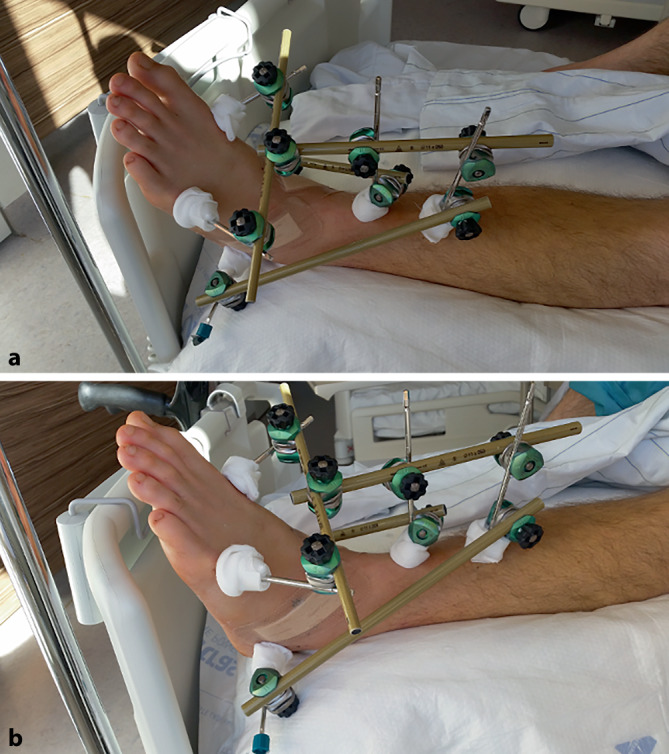
Operative Versorgung des linken Sprunggelenks mittels Fixateur externe. **a** Fixateur externe von oben, **b** Fixateur externe von lateral. Fixierung des linken Sprunggelenks in 90° durch 2 tibiale Pins, jeweils ein subkapitaler Pin in die Ossa metatarsalia 1 und 5, sowie einen Pin im Corpus calcanei mit Verbindung über zwei Etagen

Im OP erfolgten ein geschlossenes Repositionsmanöver unter Durchleuchtungskontrolle sowie additiver Applikation von 2 Kalkaneus-Pins (4,0 mm) sowie eines Talus-Pins (4,0 mm). Bei ausbleibendem Repositionserfolg wurde zum offenen Vorgehen konvertiert. Der erste Zugang wurde ventromedial über dem prominenten Talus gewählt, um das Talokalkanear- und das Talonavikulargelenk einzusehen. Der zweite Zugang wurde ventrolateral gesetzt. Die ligamentären Strukturen des unteren sowie partiell auch des oberen Sprunggelenks waren erwartungsgemäß medial nahezu vollständig und lateral partiell rupturiert. Nun konnte eine tendinöse Interposition (Sehnen des M. tibialis posterior und des M. flexor digitorum pedis longus) zwischen Talus und Kalkaneus als Repositionshindernis identifiziert werden. Nach Hervorluxieren der Sehnen über die Taluskante konnte der Kalkaneus erfolgreich reponiert werden. Die anschließende Durchleuchtungskontrolle zeigte eine scheinbar anatomische Position des Kalkaneus gegenüber dem Talus, bei jedoch persistierender Dehiszenz zwischen Talus und Os naviculare. Ursächlich hierfür war eine laterale Fehlrotation des Talus um 90° (vermutlich im Rahmen der geschlossenen Reposition entstanden), welche mechanisch behoben wurde. Auch im Chopard-Gelenk waren die ligamentären Verbindungen komplett rupturiert. Es erfolgte die Retention talonavikular mittels 2 Kirschner-Drähten (je 1,8 mm) und anschließender Stabilisierung des Sprunggelenks mittels Fixateur externe (Abb. 3). Hierzu wurden 2 tibiale Pins sowie jeweils ein subkapitaler Pin in die Ossa metatarsalia 1 und 5 eingebracht und mit dem im Corpus calcanei befindlichen Pin mittels Carbonstangen zweietagig verbunden. Das Sprunggelenk wurde somit in Neutralstellung (90°) fixiert (Abb. 4).

Die anschließende Durchleuchtungskontrolle zeigte nun eine Aufhebung der Fehlstellung sowohl im Talokalkaneargelenk als auch im Talonavikulargelenk. Auf eine Rekonstruktion der multiplen ligamentären Verletzungen wurde initial bewusst verzichtet, um eine Optimierung der operativen Rahmenbedingungen sicherzustellen (Tageszeit/Personal etc.) und die ohnehin schon fortgeschrittene Operationsdauer zu begrenzen. Es erfolgten eine Kapselnaht sowie eine Naht des Retinaculum extensorum inferius. Das Hautkolorit erholte sich rasch durch die Aufhebung der Gelenkfehlstellung und die peripheren Pulse waren tastbar.

### Verlauf

Im stationären Verlauf zeigte sich der Patient unter Ruhigstellung im Fixateur externe und einer adaptierten Analgesie schmerzarm und konnte an Unterarmgehstützen links entlastend mobilisiert werden. Es wurde eine kalkulierte Antibiotikatherapie mit 3‑mal 3 g Unacid (i.v.) für 7 Tage durchgeführt. Die Infektparameter waren während des gesamten stationären Aufenthalts im Normbereich. Die Wundverhältnisse waren allzeit unauffällig, und die Kontrollröntgenbilder zeigten eine weiterhin anatomische Stellung sowohl im Talokalkanear‑, Talonavikular- und Kalkaneokuboidalgelenk. Der Patient berichtete weiterhin über Sensibilitätsstörungen im Bereich des Vorfußes, v. a. an der 2. bis 4. Zehe. Diese waren jedoch im Verlauf rückläufig.

Nach ausgiebigem Abwägen von Pro und Contra einer Zweitoperation zur ligamentären Rekonstruktion erfolgte letztlich der Entschluss, einen „second hit“ zu vermeiden und den Fixateur atypischerweise für 12 Wochen zu belassen, um eine hinreichende Konsolidierung der Kapsel-Band-Strukturen zu erreichen. Die umliegenden Strukturen wurden physiotherapeutisch beübt, ebenso erfolgte manuelle Lymphdrainage.

**Abb. 5:**
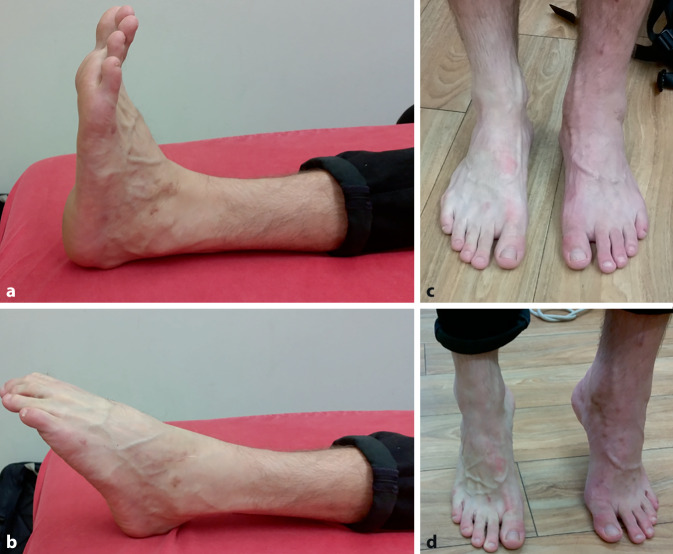
Beweglichkeit und Funktion im linken Sprunggelenk 8 Wochen nach Materialentfernung (5 Monate nach dem Trauma). **a** Maximale Dorsalextension, **b** maximale Plantarflexion, **c** Fußstellung bei normalem Stand, **d** Zehenstand beidseits

Nach 12 Wochen wurde der Fixateur externe entfernt, und es wurde sofort mit einer krankengymnastischen Beübung unter Teilbelastung mit halbem Körpergewicht begonnen. Nach ca. 3 Wochen konnte der Patient ohne Unterarmgehstützen gehen und begann sukzessive mit dem Kraftaufbau sowie längeren Gehstrecken. Insgesamt wurden 18 Einheiten Physiotherapie verordnet. Acht Wochen nach der Materialentfernung bestanden eine gute Beweglichkeit und Kraftentfaltung im Sprunggelenk (Abb. 5). Im Rahmen einer Verlaufskontrolle 7,5 Monate nach dem Trauma berichtete der Patient, dass er das linke Sprunggelenk wieder vollständig belasten könne und bereits 6 Monate nach dem Trauma resp. operativer Versorgung wieder jogge und klettere. Es bestand ein Bewegungsumfang von 10/0/40° Dorsalextension/Plantarflexion im linken Sprunggelenk sowie 15/0/10° Supination/Pronation bei sehr guter Kraftentfaltung auch im Zehenstand. Eine 10 Monate postoperativ durchgeführte Röntgenkontrolle zeigte eine gute Remineralisierung der Knochen; bis auf kleinere posttraumatische Ossifikationen im Bereich der Weichteile waren keine höhergradigen degenerativen Veränderungen nachweisbar.

Nach mittlerweile 30 Monaten klinischem Nachbeobachtungszeitraum zeigte der Patient weiterhin eine sehr gute Beweglichkeit und Belastbarkeit im betroffenen Gelenk und gab keine Schmerzen oder andere Beschwerden an. In der MRT-Untersuchung nach 30 Monaten zeigten sich tiefgreifende Knorpelabnutzungen und angrenzend subchondrale Zysten mit flauen Ödemzonen im Taluskopf sowie im hinteren unteren Sprunggelenk lateral.

## Diskussion

In diesem Fallbeispiel wird ein Patient vorgestellt, der sich bei einem Klettersturz mit Anpralltrauma gegen die Wand eine komplizierte Luxation im Bereich des linken Sprunggelenks ohne relevante Fraktur zuzog (die knöchernen Abscherungen bzw. knöchernen Bandausrisse wurden hier vernachlässigt). Erst im Rahmen der operativen Exploration konnte das volle Ausmaß der Verletzung beurteilt werden. Hierbei zeigte sich, dass es sich um eine komplexe mehrdimensionale Luxation im Talokalkaneargelenk mit Luxation des Kalkaneus im Bezug zum Talus nach lateral, kranial und ventral handelte. Weiterhin war der Talus nach plantar abgekippt, und sowohl das Talonavikulargelenk als auch das Kalkaneokuboidalgelenk waren luxiert. Beim Unfallhergang könnten die Kletterschuhe, die unser Patient getragen hat, eine Rolle gespielt haben.

Klettern und Bouldern sind Sportarten, die auch in Deutschland immer mehr an Popularität gewinnen [1, 2, 5]. Hierbei kann es besonders zu Verletzungen der oberen Extremität und der Füße kommen. Kletterschuhe mit Downturn, Vorspannung und Asymmetrie werden designed, damit Kletterer bessere Leistungen erzielen können. Jedoch wurde in den letzten Jahren vermehrt gezeigt, dass spezielle Kletterschuhe mit einem erhöhten Risiko für akute und chronische Verletzungen einhergehen. Die Mehrzahl der Kletterer trägt beim Klettern Schuhe, die kleiner sind als ihre Alltagsschuhe [4]. Die erhöhte Verletzungsstärke könnte durch die veränderte Biomechanik und Kraftverteilung in Fuß und Sprunggelenk erklärt werden. Bei unserem Patienten könnte das enge Schuhwerk die Flexibilität des Sprunggelenks eingeschränkt haben, wodurch eine starre Fixierung des Kalkaneus im Schuhwerk die Luxation begünstigt haben könnte. Ebenfalls ursächlich für die Schwere der Verletzung ist der Impact durch den Anprall gegen die Wand bei relativ niedriger Sturzhöhe. Je weiter der Sturz, desto mehr Seildehnung steht zur Verfügung, um diesen „weich“ abzufangen. Bei kurzer Sturzlänge fällt der Impact gegen die Wand deutlich höher aus, was zu schweren Anpralltraumata führt [5, 7, 8]. Typischerweise finden sich bei solchen Verletzungen jedoch Kalkaneus- und/oder Talusfrakturen [7, 8].

Bei der Literaturrecherche ist keine ähnliche Behandlung eines derartigen Falls gefunden worden. In der Vergangenheit wurden zwar einzelne Fälle beschrieben, die über subtalare Luxationen ohne begleitende Fraktur und deren Versorgung berichten, allerdings variierte insbesondere die postoperative Therapie.

Als subtalare Luxationen werden simultane Dislokationen vom Talokalkanear- und Talonavikulargelenk bezeichnet. Je nach Dislokationsrichtung des Fußes gegenüber dem Talus können sie in laterale, mediale, anteriore und posteriore Dislokationen klassifiziert werden [9–11]. Es handelt sich dabei um seltene Verletzungen, die 1–2 % aller Luxationen ausmachen [12]. Die Versorgung von subtalaren Dislokationen wurde in mehreren Fallbeispielen beschrieben und besteht meistens aus einer geschlossenen Reposition unter Anästhesie mit anschließender Ruhigstellung [13–16]. Wang et al. sowie Giannoulis et al. beschrieben die geschlossene Reposition mit anschließender Ruhigstellung in einer kurzen Unterschenkelschiene für 4 Wochen, mit anschließender Durchführung eines Mobilisationsprotokolls [13, 14]. La Palma et al. behandelten 30 Patienten mittels geschlossener Reposition und Immobilisation in einem langen Gips für 35 Tage [17]. Alternativ kann der Wechsel von einem Gips auf einen Verbandstiefel die prologierte Immobilisation sicherstellen. Verbandstiefel sind in der Regel leichter als ein klassischer Gipsverband, was den Tragekomfort erhöht und die Belastung für den Patienten reduziert. Weitere Vorteile sind eine bessere Hautverträglichkeit, da der Stiefel im Vergleich zum Gips weniger zu Hautirritationen oder Druckstellen führt. Darüber hinaus kann er bei Bedarf abgelegt werden, beispielsweise für kontrollierte Bewegungsübungen oder hygienische Maßnahmen, was insbesondere bei längerer Immobilisation von Vorteil ist.

Alternativ wurde die initiale Behandlung mittels Fixateur externe beschrieben. Milenkovic et al. konnten 11 Patienten mit offener subtalarer Luxation mittels offener Reposition und Retention im Fixateur externe über 4 bis 6 Wochen zufriedenstellend behandeln [18]. Veltman et al. präsentierten einen Fall einer offenen subtalaren Luxation, der mittels K‑Drähten und Fixateur Anlage für 8 Wochen behandelt wurde. Hier zeigten sich konkordant zu unserer Fallbeschreibung nach 12 Wochen keine Schmerzen und Bewegungseinschränkung. Der beschriebene Patient musste jedoch bei langer Belastung noch Gehhilfen nutzen und zeigte radiographisch Zeichen einer avaskulären Nekrose. Hoexum et al. beschrieben die Versorgung von zwei Patienten mit offener Reposition und anschließender interner Fixierung sowie 6 Wochen Immobilisation [19]. Die Versorgung subtalarer Luxationen ist jedoch auch mit Komplikationen verbunden. Bibbo et al. konnten in einer Fallserie zeigen, dass eine geschlossene Reposition des Subtalargelenks in 32 % der Fälle nicht möglich war [10]. Bei geeigneter und rechtzeitiger Versorgung ist ein gutes Outcome zu erwarten [16, 17, 20], jedoch werden im Verlauf auch eine eingeschränkte Mobilität sowie die Ausbildung einer subtalaren oder talonavikularen Arthrose beschrieben [16].

In dem von uns berichteten Fall konnte keine geschlossene Reposition durchgeführt werden, da sich insbesondere die Sehneninterposition zwischen Talus und Kalkaneus bzw. Os naviculare als Repositionshindernis darstellte. Daher wurde eine offene Reposition mit anschließender Ruhigstellung des Sprunggelenks in Neutralstellung mittels Fixateur externe sowie K‑Draht-Fixation im Talonavikulargelenk durchgeführt. Da wir in Würdigung der primären intraoperativen Situation bewusst auf einen Sekundäreingriff verzichteten, erfolgte der Entschluss zur atypisch langen Immobilisation im Fixateur externe mit dem Risiko einer permanenten Bewegungseinschränkung des Sprunggelenks. Es zeigten sich postoperativ keine Komplikationen und durch physiotherapeutische Beübung sowie sicherlich auch gute Patienten-Compliance und -motivation ein hervorragendes postoperatives Ergebnis mit normgerechter Beweglichkeit in OSG und USG bereits 2 Monate nach Materialentfernung. Des Weiteren zeigte sich 6 Monate nach dem Trauma eine sehr gute allgemeine und sportliche Belastbarkeit im betroffenen Sprunggelenk.

## Fazit für die Praxis

Komplexe, mehrdimensionale Luxationen des Sprunggelenks sind seltene und schwerwiegende Verletzungen, die eine umfangreiche Behandlung benötigen. Eine prolongierte Ruhigstellung mit Fixateur externe zeigte in unserem Fall sehr gute funktionelle Ergebnisse. Weitere Beobachtungsstudien sind notwendig, um die beschriebenen Ergebnisse zu bestätigen. Diese sollten wegen der geringen Fallzahlen idealerweise als Multizenterstudie durchgeführt werden.
